# Supplementary figures and images for: Distribution of heavy metal resistance elements in Canadian Salmonella 4,[5],12:i:- populations and association with the monophasic genotypes and phenotype
Source: PLoS One. 2020 Jul 27;15(7):e0236436. doi: 10.1371/journal.pone.0236436 (PMC7384650; doi:10.1371/journal.pone.0236436)

PNCS014848, PNCS014873, PNCS014875

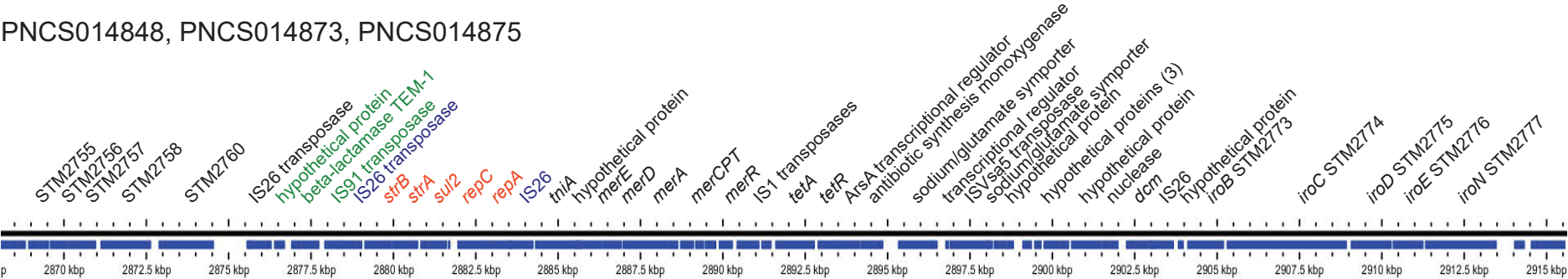

PNCS014852, PNCS014860

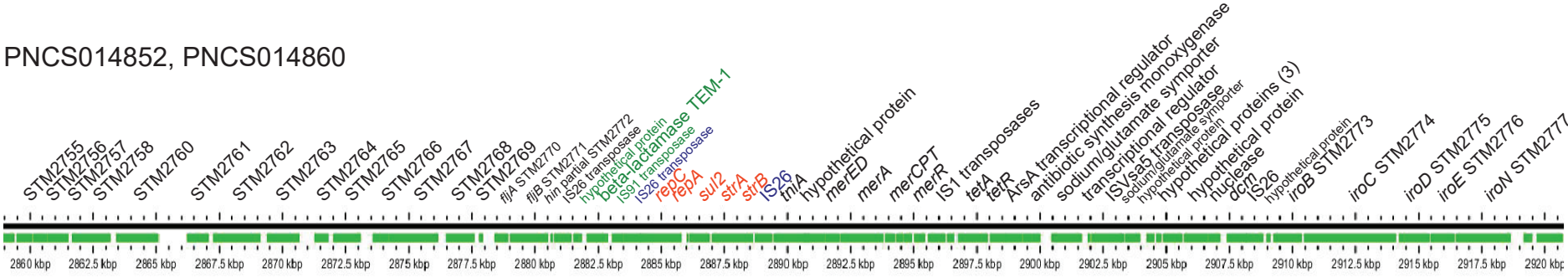

PNCS014855

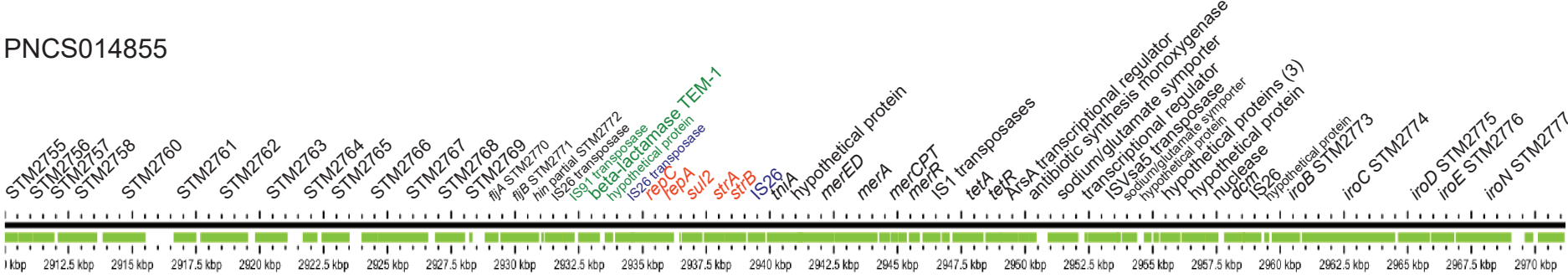

PNCS014861, PNCS014880

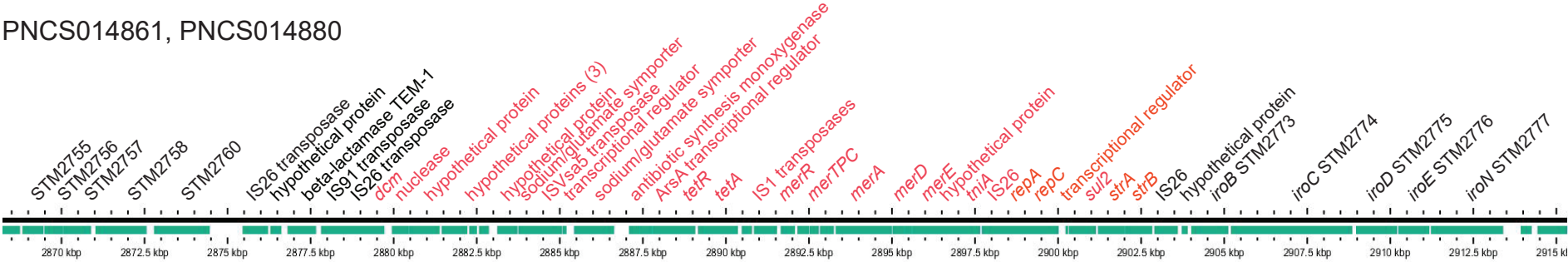

PNCS014866

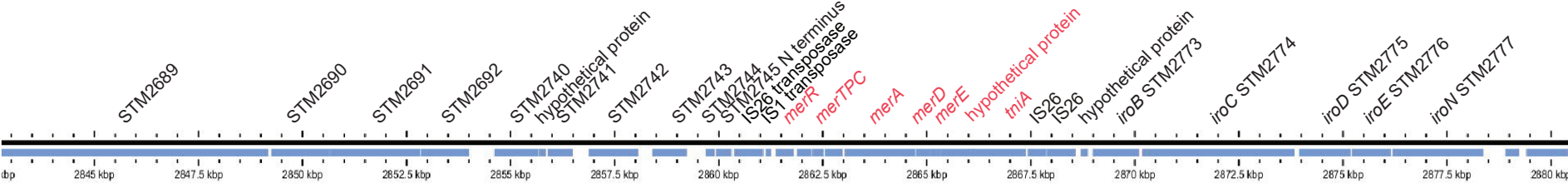

PNCS014876

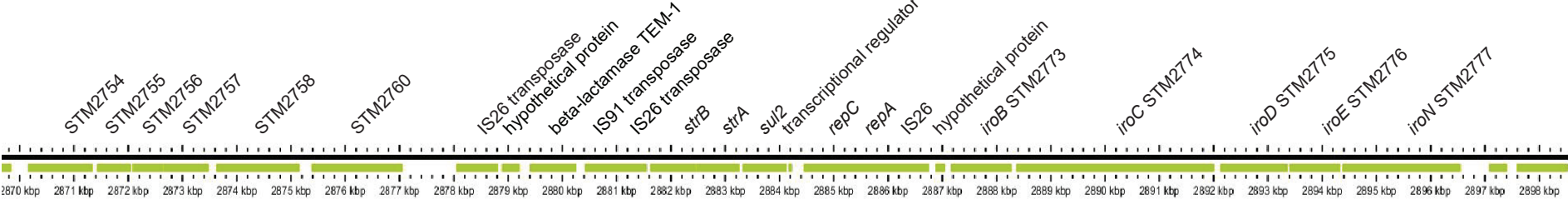

PNCS014867

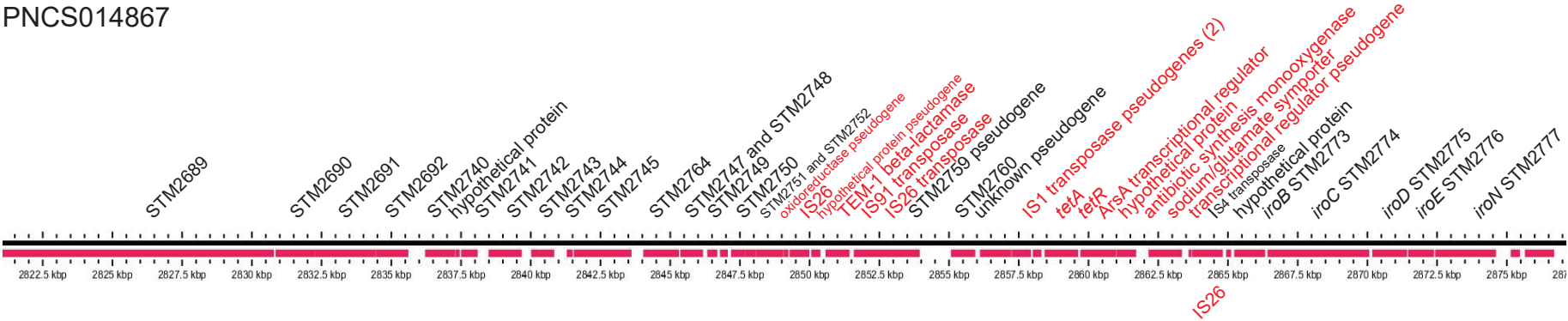

Supplement: S1 Fig — STM numbers referring to locus designations in S. Typhimurium LT2 aid in identification of the genomic location of MREL insertion. Regions associated with inversion or rearrangement are shown in red. The comparison was done using GView Server with the “pangenome analysis” settings and the figure was annotated in Adobe Illustrator CS6. (PDF) [file pone.0236436.s001.pdf]

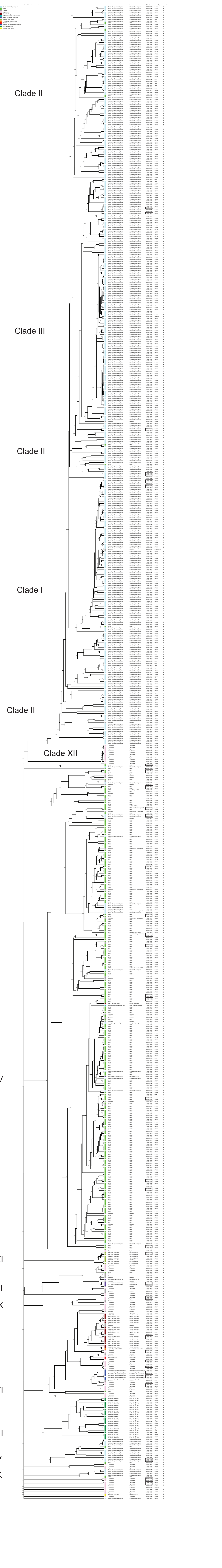

Supplement: S3 Fig — Isolates containing SGI-4 and the MREL are highlighted. Selected metadata are included. The wgMLST dendrogram was generated in BioNumerics 7.6.2. (PDF) [file pone.0236436.s003.pdf]

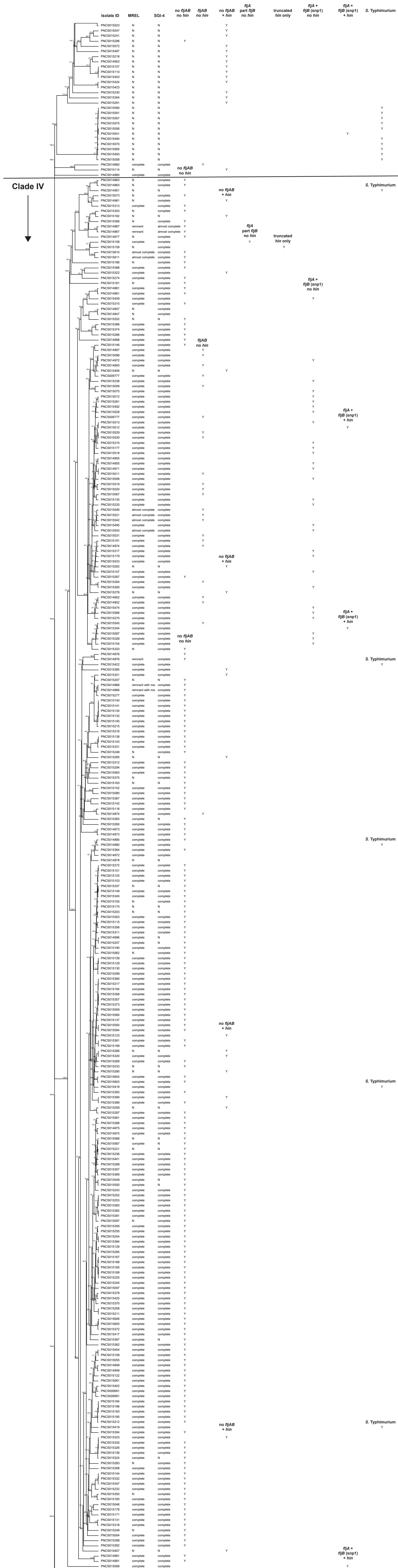

Supplement: S4 Fig — This dendrogram correlates SGI-4 and the MREL presence with fljAB hin gene presence, absence, or mutation, as well as with location in the dendrogram. The wgMLST dendrogram was generated in BioNumerics 7.6.3. (PDF) [file pone.0236436.s004.pdf]

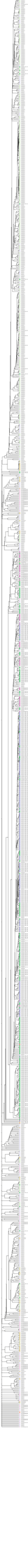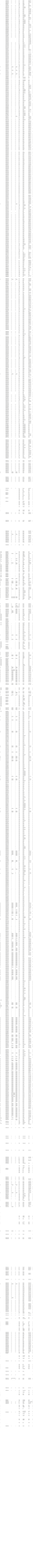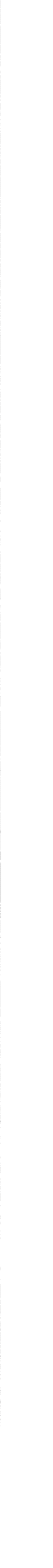

Supplement: S5 Fig — SGI-4 and MREL-containing isolates are differentiated from those without these elements. The Canadian data are from this study, while the US data are from genomes referenced in Elnekave and colleagues [14] and the Public Health England data are from genomes referenced in Petrovska and colleagues [15]. The dendrogram was produced using wgMLST data from assembled genomes using BioNumerics 7.6.3. (PDF) [file pone.0236436.s005.pdf]
