# Supplementary material for: Distribution of heavy metal resistance elements in Canadian Salmonella 4,[5],12:i:- populations and association with the monophasic genotypes and phenotype
Source: PLoS One. 2020 Jul 27;15(7):e0236436. doi: 10.1371/journal.pone.0236436 (PMC7384650; doi:10.1371/journal.pone.0236436)
Supplement: S1 File — (DOCX) [file pone.0236436.s006.docx]

Statistics for SNP analysis: outputs from the SNVPhyl pipeline

. Run ID: none

. Random seed: 0

. Subtree patterns aliasing: no

. Version: 3.3.20190321

. Time used: 0h10m31s (631 seconds)

Reference name: NC_003197.2

Total length: 4857450

Total invalid and excluded positions: 3924818

Total valid and included positions: 932631

Total valid and included positions in core genome: 542315

% of valid and included positions in core genome: 58.15%

% of all positions that are valid, included, and part of the core genome: 11.16%

Parameters and statistics for SNP phylogenetic tree: outputs from the SNVPhyl pipeline

. Sequence filename: ST-MP_SNVPhyl_200605_snvAlignment.phy

. Data set: #1

. Tree topology search: SPRs

. Initial tree: BioNJ

. Model of nucleotides substitution: GTR

. Number of taxa: 367

. Log-likelihood: -9412.57992

. Unconstrained log-likelihood: -7051.10640

. Composite log-likelihood: -537864.40455

. Parsimony: 1131

. Tree size: 1.09233

. Discrete gamma model: Yes

- Number of classes: 4

- Gamma shape parameter: 997.502

- Relative rate in class 1: 0.96005 [freq=0.250000]

- Relative rate in class 2: 0.98944 [freq=0.250000]

- Relative rate in class 3: 1.00999 [freq=0.250000]

- Relative rate in class 4: 1.04053 [freq=0.250000]

. Proportion of invariant: 0.000

. Nucleotides frequencies:

- f(A)= 0.19953

- f(C)= 0.30332

- f(G)= 0.30680

- f(T)= 0.19034

. GTR relative rate parameters :

A <-> C 0.97732

A <-> G 3.09764

A <-> T 0.82209

C <-> G 0.12876

C <-> T 3.21302

G <-> T 1.00000

. Instantaneous rate matrix :

[A---------C---------G---------T------]

-1.30932 0.27659 0.88673 0.14600

0.18195 -0.78942 0.03686 0.57061

0.57670 0.03644 -0.79073 0.17759

0.15305 0.90932 0.28626 -1.34863

Citations:

Guindon S, Dufayard JF, Lefort V, Anisimova M, Hordijk W, Gascuel O. New algorithms and methods to estimate maximum-likelihood phylogenies: assessing the performance of PhyML 3.0. Systematic Biology. 2010; 59(3): 307-321.

Guindon S, Gascuel O. A simple, fast, and accurate algorithm to estimate large phylogenies by maximum likelihood.

Systematic Biology. 2003. 52(5):696-704.
