# Supplementary material for: Distribution of heavy metal resistance elements in Canadian Salmonella 4,[5],12:i:- populations and association with the monophasic genotypes and phenotype
Source: PLoS One. 2020 Jul 27;15(7):e0236436. doi: 10.1371/journal.pone.0236436 (PMC7384650; doi:10.1371/journal.pone.0236436)
Supplement: S2 Fig — The tree was constructed using SNVPhyl and annotated in FigTree v1.4.4. It was rooted with S. Thompson strain PNCS015102 and also contained S. Heidelberg strain PNCS015430 to validate the structure. The order of annotation at each node tip is: isolate designation, province of origin, source, and genetic lesion causing the monophasic phenotype. The scale bar is at the bottom of the dendrogram. (PDF) [file pone.0236436.s002.pdf]

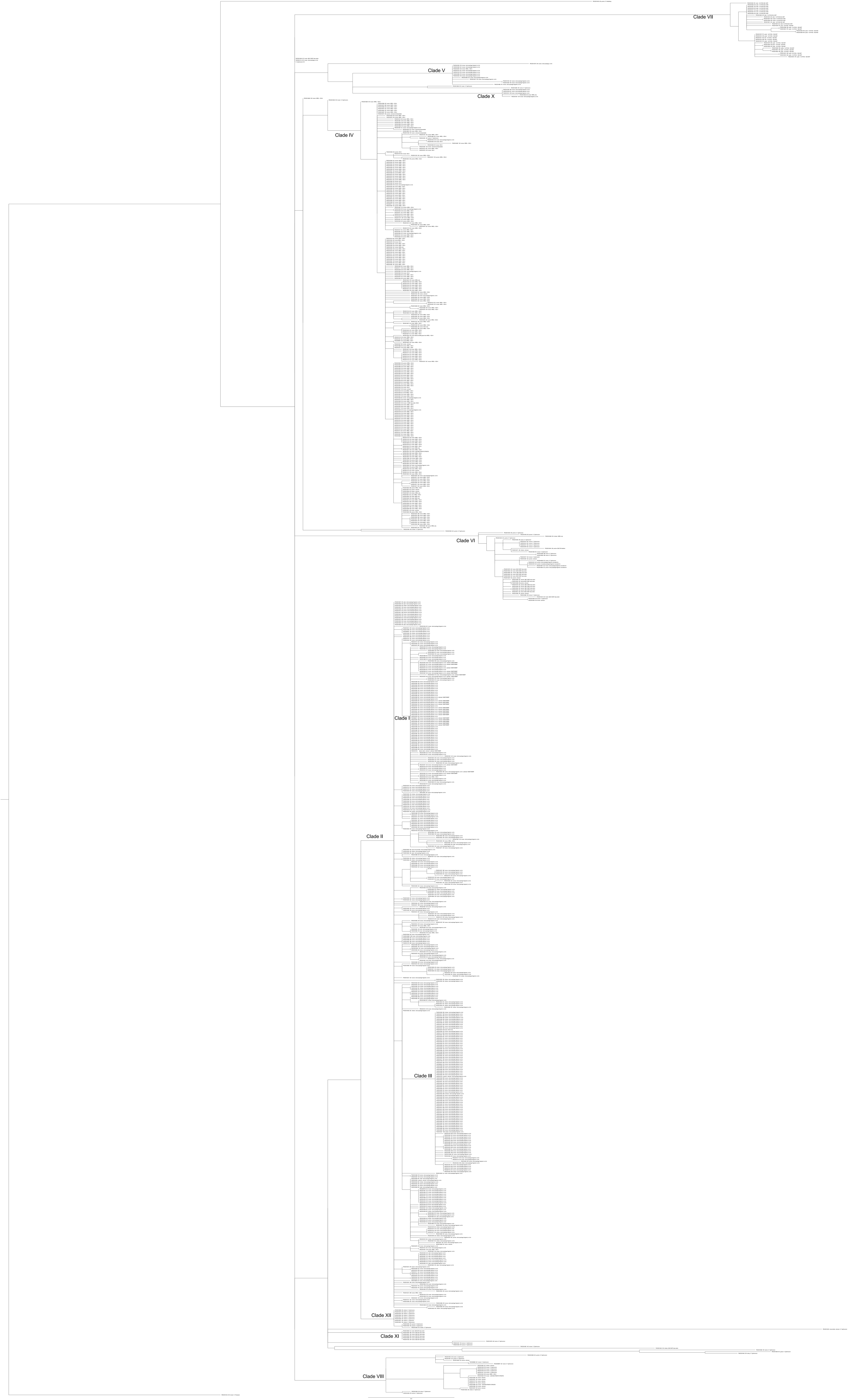

Clade VII
Clade V
Clade X
Clade IV
Clade VI
Clade I
Clade II
Clade III
Clade XII
Clade XI
Clade VIII
